# Associations of age, sex, and socioeconomic status with adherence to guideline recommendations on protein intake and micronutrient supplementation in patients with sleeve gastrectomy or Roux-en-Y gastric bypass

**DOI:** 10.1371/journal.pone.0282683

**Published:** 2023-03-03

**Authors:** Mats L. Wiese, Franziska Wilke, Simone Gärtner, Luzia Valentini, Wolfram Keßler, Ali. A. Aghdasssi, Markus M. Lerch, Antje Steveling

**Affiliations:** 1 Department of Medicine A, University Medicine Greifswald, Greifswald, Germany; 2 Institute of Evidence-based Dietetics (NIED), University of Applied Sciences Neubrandenburg, Neubrandenburg, Germany; 3 Department of General, Visceral, Thoracic and Vascular Surgery, University Medicine Greifswald, Greifswald, Germany; 4 Ludwig Maximilian University Hospital, Ludwig Maximilian University of Munich, Munich, Germany; Universitatea de Medicina si Farmacie Carol Davila Biblioteca, ROMANIA

## Abstract

**Introduction:**

Patients with bariatric surgery often show poor long-term compliance to recommendations for prevention of nutrient deficiency but it is unclear which factors contribute. We investigated the associations of age, sex, and socioeconomic status (SES) with adherence to guideline recommendations on protein intake and micronutrient supplementation.

**Methods:**

In a monocentric cross-sectional study we prospectively recruited patients with sleeve gastrectomy (SG) or Roux-en-Y gastric bypass (RYGB) and a minimum postoperative period of 6 months. Clinical and demographic data were obtained from the patients’ medical files and by questionnaire. Patients reported on supplement usage, recorded their dietary intake for seven days and underwent physical examinations including blood testing.

**Results:**

We included 35 patients (SG: n = 25, RYGB: n = 10) with a mean (+SD) postoperative period of 20.2 (±10.4) months. Distributions of age, sex and SES were comparable between the SG and RYGB groups. Non-adherence to recommended protein intake was associated with age ≥ 50 years (p = 0.041) but not sex or SES. Protein intake inversely correlated with markers of obesity. There were no significant associations of age or sex with micronutrient supplementation. Only for vitamins A (p = 0.049) and B1 (p = 0.047) higher SES was associated with greater compliance. The only manifest deficiency associated with non-adherence to micronutrient supplementation was that for folic acid (p = 0.044).

**Conclusion:**

In patients after bariatric surgery, those of older age and of lower SES might have a greater risk of unfavorable outcome and may require greater attention to micronutrient and protein supplementation.

## Introduction

For decades, obesity has been identified as a pandemic-scale health problem [1–3]. Only recently, the European Commission acknowledged obesity as a chronic relapsing disease, implying that there is no cure and lifelong treatment is mandatory [4].

Lifestyle modification and pharmacotherapy are limited in terms of sustained weight loss [5, 6] and thus not-well suited for the management of advanced obesity. By contrast, bariatric surgery has been found effective for the treatment of severe obesity [7–9]; and the number of bariatric surgeries performed globally is steadily rising [10]. A variety of surgical procedures exists that can induce different anatomical or physiological changes. The mechanisms leading to weight loss are diverse and not yet fully elucidated but restriction of food intake, malabsorption of nutrients, or a combination of both, are relevant to most procedures [11]. As a consequence, there is an increased risk of deficiency of both macro- and micronutrients which can cause severe complications, such as anemia, osteoporosis, or protein energy malnutrition [12]. Because of this inherent risk the current German S3 guideline for the ‘Surgical Treatment of Obesity and Metabolic Diseases’ recommends lifelong prophylactic nutrient supplementation for patients after bariatric surgery [13, 14]. However, several studies have shown low long-term adherence to supplementation following bariatric surgery [15–17]. Both patient- and non-patient-related factors have been suggested as causes [18]. We hypothesize that regarding patient-related factors, age, sex and socioeconomic status (SES) could be of relevance as these are known determinants of dietary intake and supplement usage in the general population [19, 20]. However, findings regarding the impact of these factors on guideline adherence in patients after bariatric surgery have been inconclusive [15, 17, 21]. To gain further knowledge on the clinical relevance of these parameters, the current study investigated the research question whether dietary intake and supplement usage among patients after bariatric surgery are associated with age, sex, and SES. In a further step, we examined the influence of guideline adherence on surrogate indicators of intermediate-term clinical outcome in order to determine the impact of nutritional recommendations and whether age, sex, and SES should be considered more thoroughly in this context.

## Materials and methods

### Study design and population

This monocentric cross-sectional study was conducted at University Medicine Greifswald, a tertiary medical center with expertise in obesity treatment located in Northeastern Germany. Between July and September 2019 all patients attending their routine postoperative care visits after bariatric surgery were approached for study participation. Patients with sleeve gastrectomy (SG) or Roux-en-Y gastric bypass (RYGB) and a postoperative period of 6 months or longer were eligible for inclusion. Wearing of a pacemaker, pregnancy, or a history of malignant or severe chronic disease were defined as exclusion criteria. The study was approved by the local institutional review board (registration no. BB 080/19) and registered at clinicaltrials.gov (NCT04587076). Written informed consent was obtained from all patients before study inclusion.

### Data collection

#### Demographic and clinical data

Patients answered a detailed questionnaire on family status, education, employment, and household income. For assessment of SES an index was calculated as described by Lampert and colleagues [22]. Briefly, this SES index comprises the three dimensions education, occupational status and equivalenced net household income, and ranges from 3–21. Scores of 3–7, 8–14, and 15–21 were categorized as low, medium, and high SES, respectively. To assess quality of life (QoL), patients additionally answered the German version of the 12-Item Short-Form Health Survey (SF-12), a validated questionnaire covering a mental and a physical component.

Clinical data on treatment modalities, postoperative body weight loss and attendance of follow-up visits were obtained from the patients’ medical files. The percentage of excess weight loss (EWL%) was calculated as follows:

$$EWL\%=\frac{Weight\;loss\;[kg]}{Excess\;weight\;[kg]}*100$$ (1)

Weightloss[kg]=Bodyweightatsurgery[kg]−Currentbodyweight[kg] (2)

Excessweight[kg]=Bodyweightatsurgery[kg]−Idealweight[kg] (3)

Formen:Idealweight[kg]=(Bodyheight[cm]−100)*0.9 (4A)

Forwomen:Idealweight[kg]=(Bodyheight[cm]−100)*0.85 (4B)

### Dietary intake and supplement usage

Patients prospectively recorded their dietary intake using a 7-day weighed dietary record prior to the study examination. Mean intake of energy, macro- and micronutrients was calculated using the OptiDiet© software version 4.2.1 (GOE, Linden, Germany). Intake of protein, vitamin, or mineral supplements was enquired from the patients, which were instructed to bring the respective packing of supplements used to the study appointment. Micronutrient supplementation among patients was subsequently compared to the German S3 guideline recommendations (S1 Table).

### Physical examinations and blood testing

Patients’ weight and height were measured with a calibrated scale and stadiometer, respectively. Subsequently, waist, hip, and mid-arm circumference were assessed using a flexible, nonelastic tape measure. We employed the Harpenden Skinfold Caliper (Baty International, West Sussex, United Kingdom) to determine triceps skinfold thickness and recorded the mean of three repeated measurements.

Body composition analysis was performed with the seca mBCA 525 (seca, Hamburg, Germany), an eight-electrode, phase-sensitive, segmental bioelectrical impedance analysis (BIA) device. Patients were instructed to restrain from eating for 4 hours, from strenuous physical activity for 12 hours, and from alcohol consumption for 24 hours; as well as to empty their bladder prior to the assessment. The measurement was conducted in a supine position using adhesive gel electrodes placed at specified anatomical sites on the dorsal surfaces of hand, wrist, ankle and foot.

Handgrip strength was tested employing the Jamar Plus+ Digital Hand Dynamometer (Patterson Medical, Warrenville, IL, USA). Three measurements were taken with the patients seated, the elbow in 90° flexion, and the wrist in a neutral position using their dominant hand. The maximum value of the three attempts was considered for analysis.

Assessment of blood pressure followed a standardized protocol employing a fully automated device (boso medicus, BOSCH+ SOHN, Jungingen, Germany). The measurement was taken in a seated position after a minimum rest of 5 minutes on the right arm.

Blood testing was performed in all patients. Selection of blood parameters that were determined was based on the guideline’s recommendations and included the following: Complete blood count, electrolytes, creatinine, blood glucose and HbA1c, vitamins A, B1, and B12, folic acid, 25-hydroxyvitamin D, parathormone, albumin, calcium, ferritin as well as zinc, copper and selenium.

### Statistical analysis

All statistical analyses were performed using IBM SPSS Statistics for Windows, version 27 (IBM Corp., Armonk, N.Y., USA). Descriptive data are presented as means (±SD) or median (IQR) for normally and non-normally distributed continuous variables, respectively. Categorical data are presented as n (%). For comparison of categorical data, the Chi-squared test or Fisher’s exact test were used. To compare continuous variables between groups, two-tailed t-test, Mann-Whitney-U test, one-way ANOVA, or Kruskal-Wallis-Test were employed as indicated by the number of groups and the distribution of the data tested. Pearson correlation coefficients were calculated to measure the degree of association between protein intake and outcome parameters. For this purpose, we performed log-transformation if data did not show normal distribution. A p-value of less than 0.05 was defined as statistically significant.

## Results

### Patient selection and characteristics

**Fig 1:**
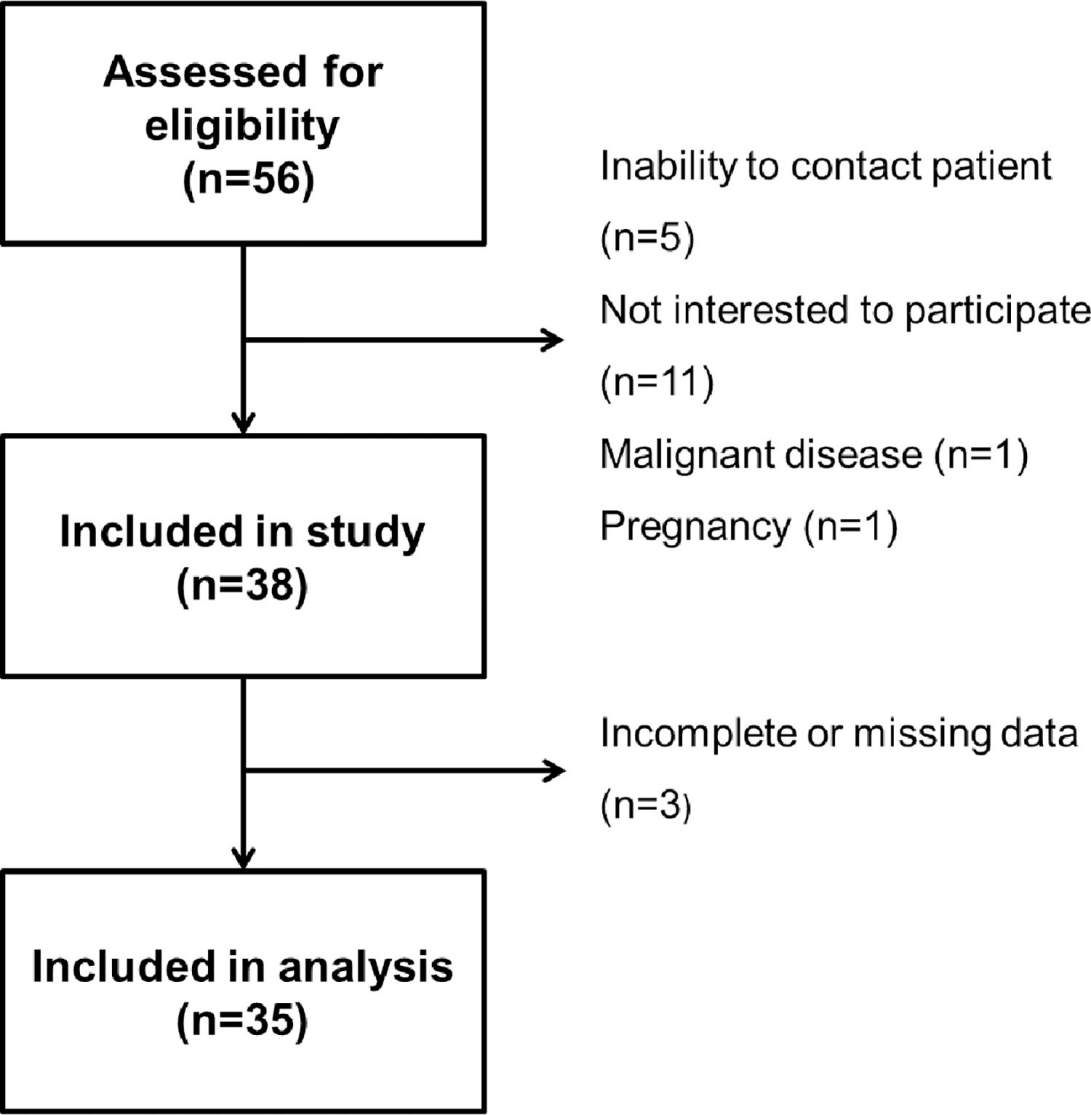
Flowchart illustrating the process of selection.

The process of patient selection is presented in Fig 1. Thirty-eight out of 56 patients initially screened for study participation were included. After removal of subjects with incomplete or missing data, 35 patients were considered in the final analysis. Between these patients and the non-participants there were no significant differences regarding age, sex, type of surgical procedure, or time post-surgery (S2 Table). Sleeve gastrectomy and RYGB had been performed in 25 and 10 subjects, respectively. Patient characteristics are summarized in Table 1. There were no significant differences between the two groups in terms of age, sex, SES, time post-surgery, smoking or marital status. The only differences were seen in patients’ present (116.5 (±28.8) kg [SG] vs. 82.7 (±14.2) kg [RYGB], p = 0.038) and pre-surgery body weight (149.8 (±30.0) kg [SG] vs. 128.0 (±16.1) kg [RYGB], p<0.001), BMI (present: 40.2 (±8.7) kg/m^2^ [SG] vs. 28.8 (±3.4) kg/m^2^ [RYGB], p<0.001; pre-surgery: 51.7 (±8.3) kg/m^2^ [SG] vs. 44.7 (±4.4) kg/m^2^ [RYGB], p = 0.018) and excess weight (present: 55.7 (±25.4) kg [SG] vs. 23.2 (±9.2) kg [RYGB], p<0.001; pre-surgery: 89.0 (±25.4) kg [SG] vs. 68.5 (±12.1) kg [RYGB], p = 0.021).

**Table 1:** Patient characteristics by bariatric surgical procedure.

		Sleeve gastrectomy (n = 25)	Roux-en-Y gastric bypass (n = 10)	p-value^a^
**Age, yrs.**		45.3 (±9.2)	49.2 (±10.2)	.274
	< 50 yrs., n (%)	18 (72)	5 (50)	.258
	≥ 50 yrs., n (%)	7 (28)	5 (50)	
**Sex**				.447
	Male, n (%)	9 (36)	2 (20)	
**Time post-surgery, months**		19.4 (±10.1)	22.0 (±11.5)	.535
**Socioeconomic status, n (%)**				.383
	Low	11 (44)	2 (20)	
	Medium	10 (40)	5 (50)	
	High	4 (16)	3 (30)	
**Active smokers, n (%)**		7 (28)	4 (40)	.689
**Marital status, n (%)**				.444
	Single	13 (52)	4 (40)	
	Married	11 (44)	5 (50)	
	Separated or divorced	-	1 (10)	
	Widowed	1 (4)	-	
**Body weight, kg**				
	Pre-surgery	149.8 (±30.0)	128.0 (±16.1)	< .001
	Present	116.5 (±28.8)	82.7 (±14.2)	.038
**Body mass index, kg/m** ^**2**^				
	Pre-surgery	51.7 (±8.3)	44.7 (±4.4)	.018
	Present	40.2 (±8.7)	28.8 (±3.4)	< .001
**Excess weight, kg**				
	Pre-surgery	89.0 (±25.4)	68.5 (±12.1)	.021
	Present	55.7 (±25.4)	23.2 (±9.2)	< .001

Data is presented as mean (±SD) unless indicated otherwise

^a^ Differences between groups were tested using two-tailed t-test for normally distributed continuous variables and Fisher’s exact test for categorical variables

### Dietary intake by surgical procedure

For energy and most nutrients, intake did not differ between patients with SG and RYGB (S3 Table). The only differences were seen for absolute intakes of carbohydrates (167.1 (±67.1) g/d [SG] vs. 130.8 (±34.0) g/d [RYGB], p = 0.043) and fat (76.1 (±32.8) g/d [SG] vs. 53.1 (±21.5) g/d [RYGB], p = 0.049) as well as nutrient density for dietary fiber (8.8 (±3.3) g/1000 kcal [SG] vs. 11.7 (±2.8) g/1000 kcal [RYGB], p = 0.018). There were no significant differences in intake of protein or micronutrients. However, median (IQR) intake of protein was less than 60 g/d only in the RYGB but not the SG group (77.8 (42.3) g/d [SG] vs. 53.6 (50.3) g/d [RYGB], p = 0.439). Five patients (14%) reported at least occasional use of protein supplements with a median (IQR) contribution to total protein intake of 8 (23) g/d in these subjects. The number of protein supplement users was comparable between the two surgical procedures (n = 3 [SG] vs. n = 2 [RYGB], p = 0.610).

### Attendance of follow-up visits

Overall attendance to follow-up visits as recommended by the S3 guideline was 81.4% (S4 Table). Lower attendance of around 70% was seen for 1-month and 3-months visits, whereas rates increased to about 90% or higher with longer postoperative period.

### Adherence to guideline recommendations by age, sex and socioeconomic status

The associations of age, sex, and SES with adherence to the S3 guideline recommendations are summarized in Table 2. We found a significant association between protein intake and age. In patients younger than 50 years, the percentage of subjects meeting the minimum daily protein intake according to the S3 guideline was 70%, compared to 25% in those who were 50 years or older (p = 0.030). This association subsisted also when considering subjects meeting the ideal recommended intake (> 1.5 g/kg normal body weight), who accounted for 17% in the younger and 8% in the older patients, as a separate group (p = 0.041). In contrast, sex and SES were not associated with protein intake. Regarding micronutrient supplementation, there were significant associations between SES and two vitamins. For vitamin A and B1, a higher percentage of subjects adhering to the recommendations was seen with increasing SES (p = 0.049 and p = 0.047, respectively). No patient with low SES followed the recommended supplementation schemes for these vitamins as compared to 43% and 57% in patients with high SES for vitamin A and B1. Supplementation of other vitamins as well as all minerals was not associated neither with SES nor age or sex. In subsequent analyses, we also tested the relation between the single dimensions of SES and adherence to guideline recommendations. None of these were individually associated with meeting recommendations on protein intake or micronutrient supplementation.

**Table 2:** Adherence to the German S3 guideline recommendations on protein intake and micronutrient supplementation in patients after bariatric surgery overall and by subgroups.

	Total	Age			Sex			Socioeconomic status			
	(n = 35)	< 50 yrs. (n = 23)	≥ 50 yrs. (n = 12)	p-value^a^	Male (n = 11)	Female (n = 24)	p-value^a^	Low (n = 13)	Medium (n = 15)	High (n = 7)	p-value^a^
**Protein intake, n (%)**				.030			.493				.416
≤ 60 g/d	16 (46)	7 (30)	9 (75)		4 (36)	12 (50)		8 (62)	5 (33)	3 (43)	
> 60 g/d	19 (54)	16 (70)	3 (25)		7 (64)	12 (50)		5 (39)	10 (67)	4 (57)	
**Micronutrient supplementation, n (%)** ^**b**^											
Vitamin A	3 (9); 18 (51); 14 (40)	3 (13); 12 (52); 8 (35)	0 (0); 6 (50); 6 (50)	.583	0 (0); 4 (36); 7 (64)	3 (13); 14 (58); 7 (29)	.145	0 (0); 6 (46); 7 (54)	0 (0); 9 (60); 6 (40)	3 (43); 2 (29); 2 (29)	.049
Vitamin D	11 (31); 19 (54); 5 (14)	5 (22); 14 (61); 4 (17)	6 (50); 5 (42); 1 (8)	.297	3 (27); 5 (45); 3 (27)	8 (33); 14 (58); 2 (8)	.413	3 (23); 8 (62); 2 (15)	4 (27); 9 (60); 2 (13)	4 (57); 2 (29); 1 (14)	.559
Vitamin E	4 (11); 18 (51); 13 (37)	4 (17); 12 (52); 7 (30)	0 (0); 6 (50); 6 (50)	.357	1 (9); 4 (36); 6 (55)	3 (13); 14 (58); 7 (29)	.347	0 (0); 7 (54); 6 (46)	1 (7); 9 (60); 5 (33)	3 (43); 2 (29); 2 (29)	.125
Vitamin B1	6 (17); 15 (43); 14 (40)	5 (22); 11 (48); 7 (30)	1 (8); 4 (33); 7 (58)	.322	2 (18); 6 (55); 3 (27)	4 (17); 12 (50); 8 (33)	.393	0 (0); 7 (54); 6 (46)	2 (13); 7 (47); 6 (40)	4 (57); 1 (14); 2 (29)	.047
Vitamin B12	35 (100); 0 (0); 0 (0)	23 (100); 0 (0); 0 (0)	12 (100); 0 (0); 0 (0)	-	11 (100); 0 (0); 0 (0)	24 (100); 0 (0); 0 (0)	-	13 (100); 0 (0); 0 (0)	15 (100); 0 (0); 0 (0)	7 (100); 0 (0); 0 (0)	-
Folic acid	7 (20); 15 (43); 13 (37)	4 (17); 12 (52); 7 (30)	3 (25); 3 (25); 6 (50)	.271	2 (18); 3 (27); 6 (55)	5 (21); 12 (50); 7 (29)	.404	0 (0); 7 (54); 6 (46)	4 (27); 6 (40); 5 (33)	3 (43); 2 (29); 2 (29)	.181
Calcium	1 (3); 21 (60); 13 (37)	1 (4); 14 (61); 8 (35)	0 (0); 7 (58); 5 (42)	1.000	0 (0); 5 (45); 6 (55)	1 (4); 16 (67); 7 (29)	.401	1 (8); 6 (46); 6 (46)	0 (0); 9 (60); 6 (40)	0 (0); 6 (86); 1 (14)	.341
Magnesium	2 (6); 20 (57); 13 (37)	1 (4); 14 (61); 8 (35)	1 (8); 6 (50); 5 (42)	.867	1 (9); 5 (45); 5 (45);	1 (4); 15 (63); 8 (33)	.508	0 (0); 8 (62); 5 (38)	2 (13); 7 (47); 6 (40)	0 (0); 5 (71); 2 (29)	.715
Iron	2 (6); 14 (40); 19 (54)	2 (9); 9 (39); 12 (52)	0 (0); 5 (42); 7 (58)	.870	0 (0); 2 (18); 9 (82)	2 (8); 12 (50); 10 (42)	.097	0 (0); 4 (31); 9 (69)	0 (0); 7 (47); 8 (53)	2 (29); 3 (43); 2 (29)	.105
Copper	25 (71); 7 (20); 3 (9)	18 (78); 4 (17); 1 (4)	7 (58); 3 (25); 2 (17)	.378	9 (82); 1 (9); 1 (9)	16 (67); 6 (25); 2 (8)	.716	11 (85); 1 (8); 1 (8)	10 (67); 4 (27); 1 (7)	4 (57); 2 (29); 1 (14)	.652
Zinc	3 (9); 17 (49); 15 (43)	3 (13); 11 (48); 9 (39)	0 (0); 6 (50); 6 (50)	.660	0 (0); 4 (36); 7 (64)	3 (13); 13 (54); 8 (33)	.216	1 (8); 6 (46); 6 (46);	0 (0); 8 (53); 7 (47)	2 (29); 3 (43); 2 (29)	.390
Selenium	27 (77); 6 (17); 2 (6)	19 (83); 4 (17); 0 (0)	8 (67); 2 (17); 2 (17)	.246	9 (82); 1 (9); 1 (9)	18 (75); 5 (21); 1 (4)	.672	11 (85); 1 (8); 1 (8)	11(73); 4 (27); 0 (0)	5 (71); 1 (14); 1 (14)	.465

^a^ Significant differences between subgroups were tested using Fisher’s exact test

^b^ Data is presented as number and percentage of patients following recommended, other than recommended, or no supplementation

### Protein intake and clinical outcome parameters

**Fig 2:**
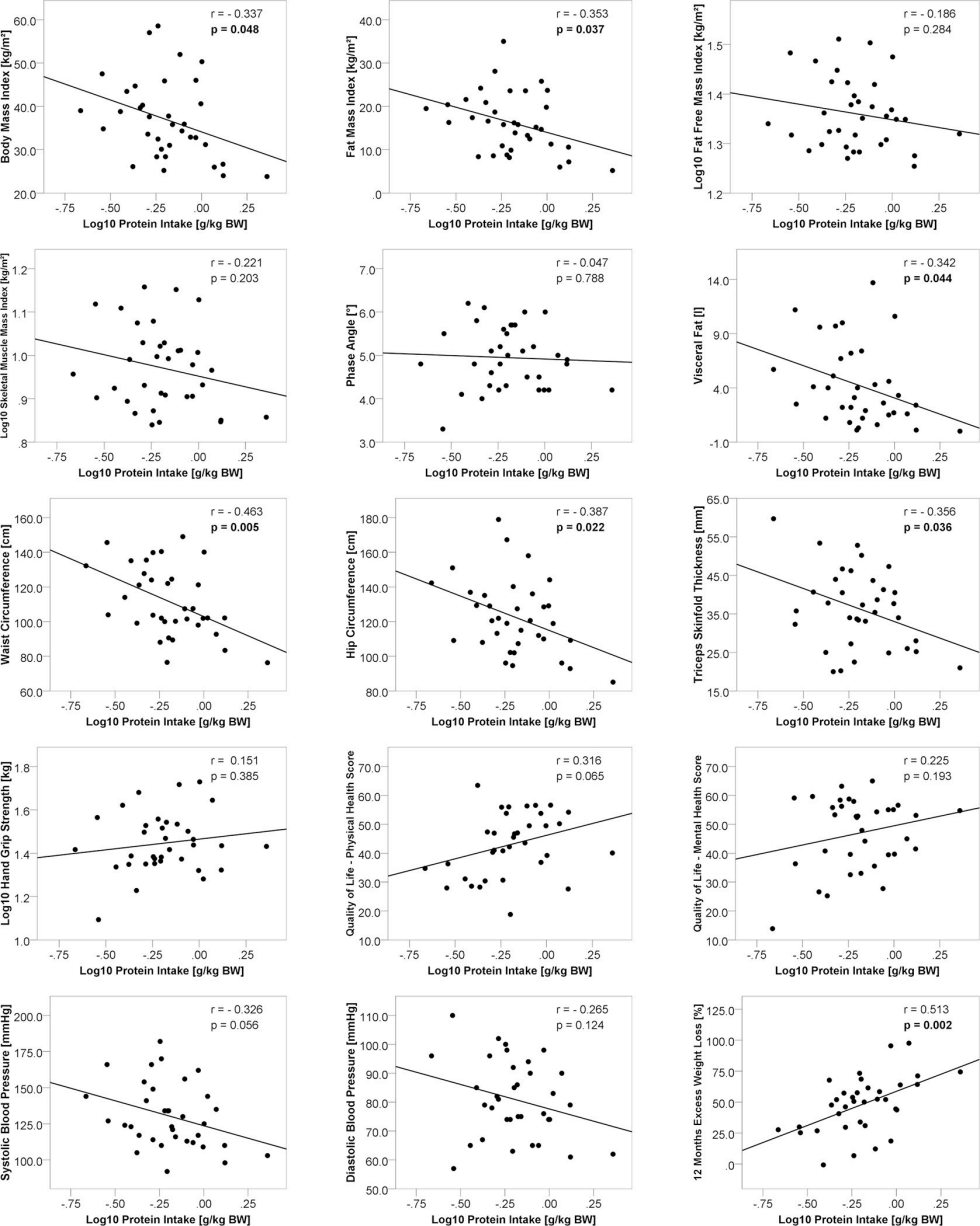
Pearson correlation between protein intake and clinical outcome parameters.

We found no significant associations between adherence to the guideline recommendations for protein intake, neither minimum (> 60 g/d) nor ideal (> 1.5 g/ kg of normal body weight), and clinical outcome parameters (Table 3). However, there was a significant inverse correlation between protein intake per kg of current body weight and visceral body fat (r = -0.342, p = 0.044), as well as with several other obesity-related parameters, including BMI (r = -0.337, p = 0.048), fat mass index (r = -0.353, p = 0.037), triceps skinfold thickness (r = -0.356, p = 0.036), waist (r = -0.463, p = 0.005) and hip circumference (r = -0.387, p = 0.022) (Fig 2). In addition, higher protein intake also correlated with greater 12 months EWL whereas no significant correlations were seen for muscle mass, handgrip strength, blood pressure or quality of life subscales.

**Table 3:** Outcome parameters by adherence to the German S3 guideline recommendations on protein intake.

	Protein intake				
	≤ 60 g/d (n = 16)	> 60 g/d (n = 14)	> 1.5 g/kg normal body weight (n = 5)	p-value^a^	p-value^b^
**Anthropometry**					
Body mass index, kg/m^2^	35.1 (±6.8)	37.1 (±10.5)	42.5 (±11.4)	.287	.272
Waist circumference, cm	109.6 (±19.7)	111.2 (±18.1)	117.7 (±29.4)	.743	.629
Hip circumference, cm	118.6 (±17.2)	124.6 (±24.4)	129.0 (±27.4)	.583	.330
Waist-to-Hip ratio	0.92 (±0.08)	0.90 (±0.11)	0.90 (±0.07)	.830	.546
Triceps skinfold thickness, mm	34.6 (±10.93)	37.6 (±9.54)	38.0 (±10.18)	.672	.371
**Body composition**					
Fat mass index, kg/m^2^	15.2 (±5.5)	16.2 (±7.9)	19.6 (±8.3)	.473	.439
Visceral fat volume, l	2.8 (4.4)	3.0 (5.7)	4.6 (11.3)	.776	.635
Fat free mass index, kg/m^2^	18.7 (3.8)	20.6 (5.4)	20.8 (8.1)	.179	.109
Skeletal muscle mass index, kg/m^2^	8.3 (2.4)	10.0 (2.9)	10.2 (5.5)	.208	.095
**Muscle function**					
Hand grip strength, kg	24.3 (12.1)	28.2 (13.0)	27.4 (20.0)	.573	.317
**Weight loss**					
Excess weight loss after 12 months, %	44.7 (±19.8)	55.6 (±24.2)	38 (±24.7)	.257	.409
**Blood pressure**					
Systolic, mmHg	132 (±24)	130 (20)	126 (23)	.863	.671
Diastolic, mmHg	81 (±15)	81 (±12)	80 (±15)	.982	.898
**Quality of life**					
Physical health score	39.6 (±12.6)	47.5 (±9.0)	41.8 (±4.9)	.136	.090
Mental health score	52.8 (21.1)	48.7 (19.6)	54.7 (20.5)	.705	.781

Data is presented as mean (±SD) or median (IQR) for normally and non-normally distributed variables, respectively.

^a^ Differences between groups (> 60 g/d vs. ≤ 60 g/d vs. > 1.5 g/kg normal body weight) were tested using one-way ANOVA and Kruskal-Wallis-Test for normally distributed and for non-normally distributed variables, respectively.

^b^ Differences between groups (> 60 g/d vs. ≤ 60 g/d) were tested using two-tailed t-test and Mann-Whitney-U test for normally distributed and non-normally distributed variables, respectively.

### Supplementation schemes and micronutrient deficiency

Overall prevalence of manifested deficiency was low, with the exception of vitamin D which was deficient in 51% of patients (Table 4). Usage of supplements ranged from 40% for copper to 100% for vitamin B12 and tended to be higher for vitamins than for minerals. Patients, following the recommended supplementation scheme, did not show deficiency for any micronutrient except vitamin D. Conversely, manifested deficiency in other micronutrients was only observed when patients took no supplements or followed another supplementation scheme. Yet, only for folic acid a statistically significant association with categories of supplement usage was found (p = 0.044).

**Table 4:** Supplement usage and prevalence of manifested deficiency of critical micronutrients in patients after bariatric surgery (n = 35).

	Supplement usage, n (%)	Manifested deficiency, n (%)				
		Total	Recommended supplementation	Other scheme	No supplementation	p-value^a^
**Vitamin A**	21 (60)	1 (3)	0 (0)	1 (6)	0 (0)	1.000
**Vitamin D**	30 (86)	18 (51)	5 (45)	9 (47)	4 (80)	.187
**Vitamin E**	22 (63)	0 (0)	0 (0)	0 (0)	0 (0)	-
**Vitamin B1**	21 (60)	0 (0)	0 (0)	0 (0)	0 (0)	-
**Vitamin B12**	35 (100)	0 (0)	0 (0)	-	-	-
**Folic acid**	22 (63)	3 (9)	0 (0)	0 (0)	3 (23)	**.044**
**Calcium**	22 (63)	0 (0)	0 (0)	0 (0)	0 (0)	-
**Magnesium**	22 (63)	3 (9)	0 (0)	2 (10)	1 (8)	1.000
**Iron**	16 (46)	1 (3)	0 (0)	1 (7)	0 (0)	.457
**Copper**	14 (40)	0 (0)	0 (0)	0 (0)	0 (0)	-
**Zinc**	20 (57)	0 (0)	0 (0)	0 (0)	0 (0)	-
**Selenium**	20 (57)	0 (0)	0 (0)	0 (0)	0 (0)	-

^a^ Fisher’s exact test was used to determine significant differences in prevalence of manifested nutrient deficiency between supplemented and unsupplemented patients.

## Discussion

This work investigated the associations of age, sex, and SES with guideline adherence regarding intake and supplementation of critical nutrients in patients after bariatric surgery. We found that patients 50 years or older were less likely to meet recommended protein intake in comparison to those being younger. Further, we saw that higher SES was associated with greater adherence to supplementation of vitamins A and B1. Regarding the implications of guideline adherence on intermediate outcome, we could show that protein intake per kg of body weight inversely correlated with markers of obesity and non-adherence to micronutrient supplementation, partially reflected in manifested deficiency.

Our results regarding the association of age with compliance to guideline recommendations for protein intake are supported by findings in the general public. A recent meta-analysis [23] showed that even in developed countries, including Germany, inadequate protein intake in community-dwelling older subjects is not an exception, with approximately 35% consuming less than 1 g of protein per kilogram of body weight. A high prevalence of insufficient protein intake after bariatric surgery has been reported in multiple studies [24–27] and our findings suggest that higher age will likely aggravate this issue. Ultimately, insufficient protein intake imposes a risk for sarcopenia, which in two recent studies [28, 29] has been identified to be a relevant issue as early as one year after bariatric surgery, even in younger and initially non-sarcopenic patients. While primary sarcopenia is age-related, its secondary form can be caused by obesity itself or by poor nutrient intake and absorption [30]. Concurrence of higher age and multiple other risk factors therefore calls for particular caution in older patients following bariatric surgery. Although we could not show a direct association between protein intake and skeletal muscle mass or handgrip strength, higher protein intake correlated with EWL after 12 months. Moreover, we found an inverse association with several markers of obesity, including fat mass index and visceral fat volume. A beneficial effect of higher protein consumption on EWL or body composition has also been reported in other studies [24, 31–33]. In line with our results, Schollenberger and colleagues [32] showed an enhanced loss of body fat mass in patients who received a protein supplement for six months after bariatric surgery compared to placebo; but only a trend towards ameliorated loss of lean body mass and no difference in hand grip strength was seen. In addition, findings from a Swedish prospective study [33] investigating long-term outcome after bariatric surgery indicate that changing in macronutrient composition of diet towards higher protein intake instead of fat during the first six months is associated with a better 10-year weight loss. Sustained satiety, energy expenditure and sparing of lean body mass have been suggested as explanations for the beneficial effect of higher protein intake in patients with negative energy balance [34]. However, the exact mechanisms, especially in patients after bariatric surgery, require further elucidation [35].

Regarding adherence to guideline recommendations for micronutrient supplementation we only saw an association between SES, but not age and sex, and selected vitamins, specifically vitamin A and B1. Only a limited number of studies have investigated these relations before but associations were either non-significant or inconsistent results were reported [15, 17, 21]. For instance, two studies have reported an association with sex [15, 21]. However, one found better compliance in male patients [15] whereas the other showed higher adherence in females [21]. In the third study, in agreement with our own findings, no differences between sexes regarding supplement usage were seen [17]. Moreover, in line with our study a significant association with age has so far not been shown in adult populations [17, 21]. However, the poor compliance seen in adolescents, for instance, suggests that adherence to supplement intake might be an issue in particular age groups [36]. The significant association we found between SES and selected vitamins, with greater adherence in groups of higher SES, has not been shown in this context before. In a previous investigation non-adherence was associated with full-time employment, whereas a higher level of education was not associated [21]. It must be considered, though, that we analyzed SES as a composite metric, which also includes reported household income. The costs of supplementation have been reported to be an obstacle to nutrient supplement intake in patients after bariatric surgery [15]. However, in our additional analyses the individual dimensions of SES were not significantly associated with compliance suggesting an additive effect. Hence, SES as a composite metric may be more important than the individual factors and this could explain why we only saw greater adherence in patients with higher SES. The German S3 guideline recommends that vitamin A and B1 should be taken twice daily as constituents of a multivitamin supplement. We did not find an association for other vitamins, for which the same supplementation regime is recommended, e.g., vitamin E and K. This finding confirms that not all multivitamin preparations are equally suited to meet the requirements of patients with bariatric surgery and those with lower SES might favor less appropriate products because of lower level of education or a lower price of these supplements.

Overall, we saw poor adherence to the guideline recommendations on micronutrient supplementation. Yet, except for vitamin D, manifest deficiency was rare and it must be noted that comparable vitamin D levels have been observed in the general population of Northeast Germany [37]. Moreover, only omission of folic acid supplementation was associated with biochemical deficiency. Several studies found higher rates of micronutrient deficiency following SG or RYGB [38–44] but inconsistent associations with adherence to supplementation have been reported. While some studies observed manifest deficiencies irrespective of supplement intake [38, 41–43], other trials showed lower prevalence of deficiency in supplemented patients [39, 40, 44]. Caution is indicated when interpreting these findings. A recent meta-analysis [45] found that most studies reporting on micronutrient deficiency after bariatric surgery are of low quality and omit important confounding factors, such as underestimation of preoperative deficiencies and inadequate recording of supplementation adherence. Furthermore, it must be considered that recommendations on supplementation vary, for instance by country or institution. Minor deviations from recommendations will still provide better protection against deficiency than perfect adherence to inappropriate supplementation regimes. For instance, Lanzarini and colleagues [44] reported that in patients with SG or RYGB daily supplementation of 400 IU of vitamin D3 was insufficient to prevent deficiency and high dose supplementation, i.e., 16,000 IU biweekly or even higher, may be required. The German S3 guideline [13, 14] recommends meticulous postoperative care including monitoring of blood parameters and prophylactic supplementation of micronutrients. Hence, it is plausible that in our cohort overall deficiencies were rare although most patients reported to follow supplementation regimes other than recommended or not to use supplements at all. In addition, some nutrient deficiencies might manifest only after longer time periods depending on preoperative reserves [46, 47]. In consequence, our results do not suggest the recommendations for mandatory prophylactic micronutrient supplementation following SG or RYGB to be inappropriate or redundant as no substitution will ultimately result in nutrient deficiency with—in the worst case—fatal consequences [48].

There are some limitations to our study which must be acknowledged. First, we cannot entirely rule out different types of bias, including selection and social desirability bias. However, comparable characteristics of study patients and non-participants as well as observations of unsuccessful body weight loss and reported poor adherence to guideline recommendations refute this possibility. Secondly, because of the study design, assessment of outcome parameters before bariatric surgery, with exception of body weight, was not possible. Thus, there is a chance that some associations with outcome parameters might have been affected by preoperatively existing differences, although this is unlikely for most parameters. In our center, nutrient deficiencies, for instance, are thoroughly tested before surgery and thus can almost certainly be excluded as a presurgical condition. Third, we included patients at different time points after surgery which might affect analysis on some outcome parameters. The range of time post-surgery was, however, narrow and these differences more likely influence outcome parameter analysis than the association of guideline adherence with age, sex or SES, which was the primary objective of our study. Last, due to the small number of patients and the short follow-up period generalizability of our findings may be limited. Therefore, it is desirable that our results are either confirmed or refuted, preferably in the setting of a large-scale, multicenter long-term follow-up study.

In conclusion, we have shown that with regard to the unresolved determinants of dietary compliance in patients after bariatric surgery lower age and higher SES are associated with greater adherence to the German S3 guideline recommendations on protein intake and micronutrient supplementation, respectively. Protein intake inversely correlated with obesity-related parameters and poorer adherence to recommended micronutrient supplementation was in part linked to deficiency. Consequently, older patients and those with lower SES might ultimately be at higher risk of unfavorable outcome following bariatric surgery, suggesting that it may be indicated to pay special attention to these groups during follow-up in the future.

## Supporting information

S1 Table Recommendations on prophylactic micronutrient supplementation for patients with sleeve gastrectomy or Roux-en-Y gastric bypass according to the German S3 guideline for the ‘Surgical Treatment of Obesity and Metabolic Diseases’. (DOCX)

S2 Table Comparison of basic patient characteristic between participants and non-participants. (DOCX)

S3 Table Dietary intake by bariatric surgical procedure. (DOCX)

S4 Table Attendance of follow-up visits according to the S3 guideline. (DOCX)

S1 Dataset Individual patient data. (XLSX)
