# Correction: Associations of age, sex, and socioeconomic status with adherence to guideline recommendations on protein intake and micronutrient supplementation in patients with sleeve gastrectomy or Roux-en-Y gastric bypass

**DOI:** 10.1371/journal.pone.0314699

**Published:** 2024-11-26

**Authors:** Mats L. Wiese, Franziska Wilke, Simone Gärtner, Luzia Valentini, Wolfram Keßler, Ali A. Aghdassi, Markus M. Lerch, Antje Steveling

The sixth author’s name is spelled incorrectly. The correct name is: Ali A. Aghdassi. The correct citation is: Wiese ML, Wilke F, Gärtner S, Valentini L, Keßler W, Aghdassi AA, et al. (2023) Associations of age, sex, and socioeconomic status with adherence to guideline recommendations on protein intake and micronutrient supplementation in patients with sleeve gastrectomy or Roux-en-Y gastric bypass. PLOS ONE 18(3): e0282683. https://doi.org/10.1371/journal.pone.0282683
