# Supplementary material for: Associations of age, sex, and socioeconomic status with adherence to guideline recommendations on protein intake and micronutrient supplementation in patients with sleeve gastrectomy or Roux-en-Y gastric bypass
Source: PLoS One. 2023 Mar 3;18(3):e0282683. doi: 10.1371/journal.pone.0282683 (PMC9983924; doi:10.1371/journal.pone.0282683)
Supplement: S1 Table — (DOCX) [file pone.0282683.s001.docx]

**S1 Table. Recommendations on prophylactic micronutrient supplementation for patients with sleeve gastrectomy or Roux-en-Y gastric bypass according to the German S3 guideline for the ‘Surgical Treatment of Obesity and Metabolic Diseases’.**

|  | **Sleeve gastrectomy** | **Roux-en-Y gastric bypass** |
| --- | --- | --- |
| Folic acid | MVM preparation bid | 600 μg/d |
| Vitamin B1 | MVM preparation bid, no dose recommendation | |
| Vitamin B12 | p.o.: 1000 μg/d  IM: 1000 – 3000 μg/d every 3 to 6 months | |
| Vitamin A | MVM preparation bid | |
| Vitamin D | At least 3000 IU/d | |
| Vitamin E, K | MVM preparation bid, no dose recommendation | |
| Calcium citrate | 1200 – 1500 mg/d | |
| Iron sulfate, fumarate, gluconate | MVM preparation bid | 50 mg/d |
| Magnesium citrate | 200 mg/d | |
| Zinc gluconate, sulfate, acetate | MVM preparation bid | |
| Copper gluconate, oxide, sulfate  Selenium as sodium selenite | No recommendation | MVM preparation bid with 2 mg/d of copper |

modified from Dietrich et al. 2018 [13]

bid, twice a day; MVM preparation, multivitamin and mineral preparation

A MVM preparation should be chosen that is rich in micronutrients, in amounts that are within 100% of the RDA (recommended daily allowance).
