# Supplementary material for: Associations of age, sex, and socioeconomic status with adherence to guideline recommendations on protein intake and micronutrient supplementation in patients with sleeve gastrectomy or Roux-en-Y gastric bypass
Source: PLoS One. 2023 Mar 3;18(3):e0282683. doi: 10.1371/journal.pone.0282683 (PMC9983924; doi:10.1371/journal.pone.0282683)
Supplement: S2 Table — (DOCX) [file pone.0282683.s002.docx]

**S2 Table. Comparison of basic patient characteristic between participants and non-participants.**

|  | | **Participants**  **(n=35)** | **Non-participants**  **(n=18)** | p-value^a^ |
| --- | --- | --- | --- | --- |
| **Age, yrs.** | | 46.4 (±9.6) | 50.4 (±12.6) | .203 |
| **Sex** | |  |  | .888 |
|  | Male, n (%) | 11 (31) | 6 (33) |  |
| **Surgical procedure** | |  |  | .248 |
|  | Sleeve gastrectomy, n (%) | 25 (71) | 10 (56) |  |
|  | Roux-en-Y gastric bypass, n (%) | 10 (29) | 8 (44) |  |
| **Time post-surgery, months^b^** | | 17.0 (12) | 23.3 (18) | .102 |

Data is presented as mean (±SD) unless indicated otherwise

^a^ Differences between groups were tested using two-tailed t-test for normally distributed continuous variables, Mann-Whitney-U test for non-normally distributed continuous variables, and Chi-squared test for categorical variables

^b^ Data is presented as median (IQR)
