# Supplementary material for: Associations of age, sex, and socioeconomic status with adherence to guideline recommendations on protein intake and micronutrient supplementation in patients with sleeve gastrectomy or Roux-en-Y gastric bypass
Source: PLoS One. 2023 Mar 3;18(3):e0282683. doi: 10.1371/journal.pone.0282683 (PMC9983924; doi:10.1371/journal.pone.0282683)
Supplement: S3 Table — (DOCX) [file pone.0282683.s003.docx]

**S3 Table. Dietary intake by bariatric surgical procedure.**

|  | | **Sleeve gastrectomy**  **(n=25)** | **Roux-en-Y gastric bypass (n=10)** | p-value^a^ |
| --- | --- | --- | --- | --- |
| Energy, kcal/d | | 1690 (±635) | 1285 (±327) | .066 |
| Carbohydrates | |  |  |  |
|  | g/d | 167.1 (±67.1) | 130.8 (±34.0) | **.043** |
|  | % of energy | 40.4 (±7.0) | 42.8 (±10.0) | .433 |
| Fat | |  |  |  |
|  | g/d | 76.1 (±32.8) | 53.1 (±21.5) | **.049** |
|  | % of energy | 38.0 (5.0) | 37.5 (14.0) | .439 |
| Protein | |  |  |  |
|  | g/d | 77.8 (42.3) | 53.6 (50.3) | .439 |
|  | g/kg bodyweight | 0.66 (0.5) | 0.61 (0.5) | .733 |
| Sucrose | |  |  |  |
|  | g/d | 38.5 (51.7) | 29.3 (26.4) | .627 |
|  | % of energy | 9.6 (5.6) | 12.0 (6.9) | .439 |
| Dietary fiber | |  |  |  |
|  | g/d | 13.1 (6.6) | 13.1 (6.5) | .788 |
|  | g/1000 kcal | 8.8 (±3.3) | 11.7 (±2.8) | **.018** |
| Vitamin A, mg/d | | 0.36 (±0.5) | 0.38 (±0.4) | .872 |
| Vitamin D, µg/d | | 1.7 (2.0) | 0.8 (3.1) | .131 |
| Vitamin E, mg/d | | 7.9 (6.9) | 6.3 (4.7) | .653 |
| Vitamin K, µg/d | | 210 (±104) | 186 (±57) | .506 |
| Vitamin B1, mg/d | | 1.0 (1.1) | 1.1 (1.3) | .900 |
| Folic acid, mg/d | | 160 (±72) | 159 (±66) | .978 |
| Vitamin B12, mg/d | | 4.2 (±2.3) | 3.5 (±2.1) | .371 |
| Calcium, mg/d | | 900 (726) | 857 (420) | .653 |
| Magnesium, mg/d | | 325 (±126) | 326 (±138) | .994 |
| Iron, mg/d | | 8.1 (4.3) | 6.9 (6.6) | .928 |
| Copper, mg/d | | 1.45 (±0.65) | 1.64 (±0.70) | .471 |
| Zinc, mg/d | | 7.0 (3.5) | 5.8 (5.0) | .397 |

Data is presented as mean (±SD) or median (IQR) for normally and non-normally distributed variables, respectively.

Micronutrient consumption does not include intake from supplements.

^a^ Differences between groups were tested using two-tailed t-test and Mann-Whitney-U test for normally and non-normally distributed variables, respectively.
