# Supplementary material for: Associations of age, sex, and socioeconomic status with adherence to guideline recommendations on protein intake and micronutrient supplementation in patients with sleeve gastrectomy or Roux-en-Y gastric bypass
Source: PLoS One. 2023 Mar 3;18(3):e0282683. doi: 10.1371/journal.pone.0282683 (PMC9983924; doi:10.1371/journal.pone.0282683)
Supplement: S4 Table — (DOCX) [file pone.0282683.s004.docx]

**S4 Table. Attendance of follow-up visits according to the S3 guideline.**

| **Follow-up visit** | **Attended / Recommended, n/n** | **%** |
| --- | --- | --- |
| 1 month | 24/35 | 68.6 |
| 3 months | 25/35 | 71.4 |
| 6 months | 31/35 | 88.6 |
| 12 months | 24/26 | 92.3 |
| 18 months | 14/16 | 87.5 |
| 24 months | 8/9 | 88.9 |
| 36 months | 5/5 | 100.0 |
| **Total** | 131/161 | 81.4 |
